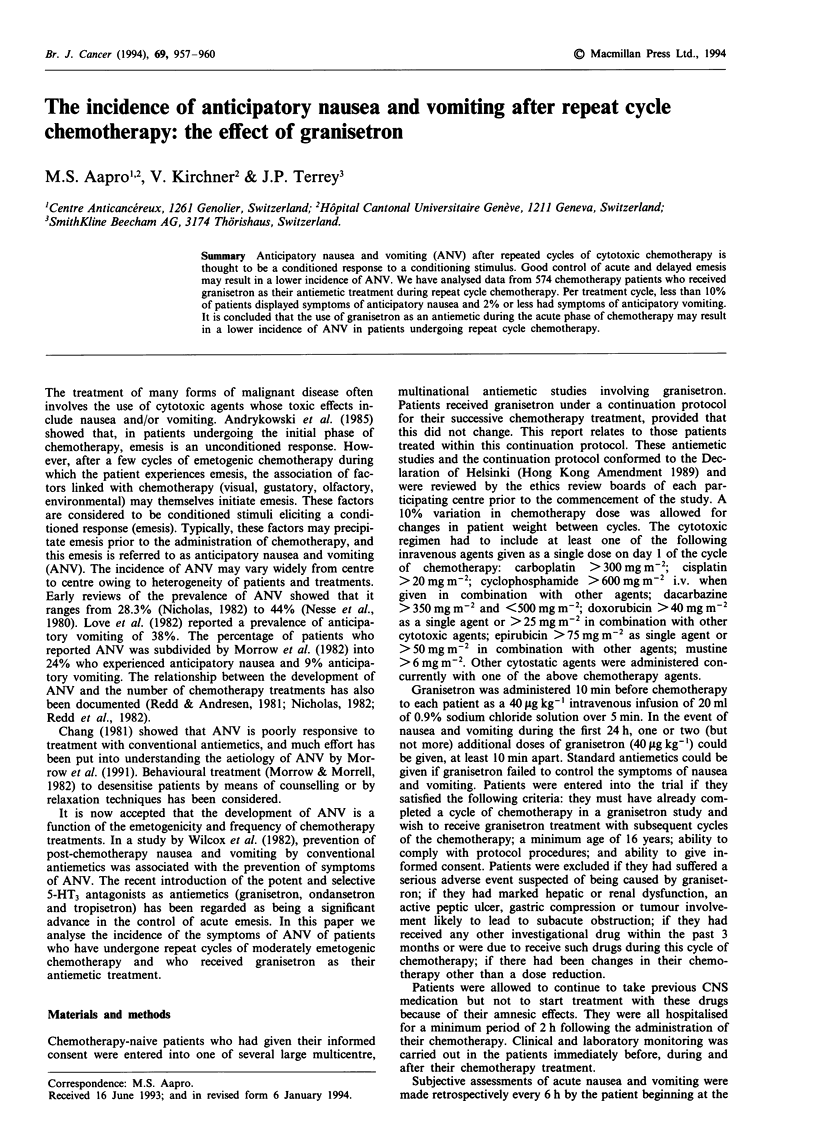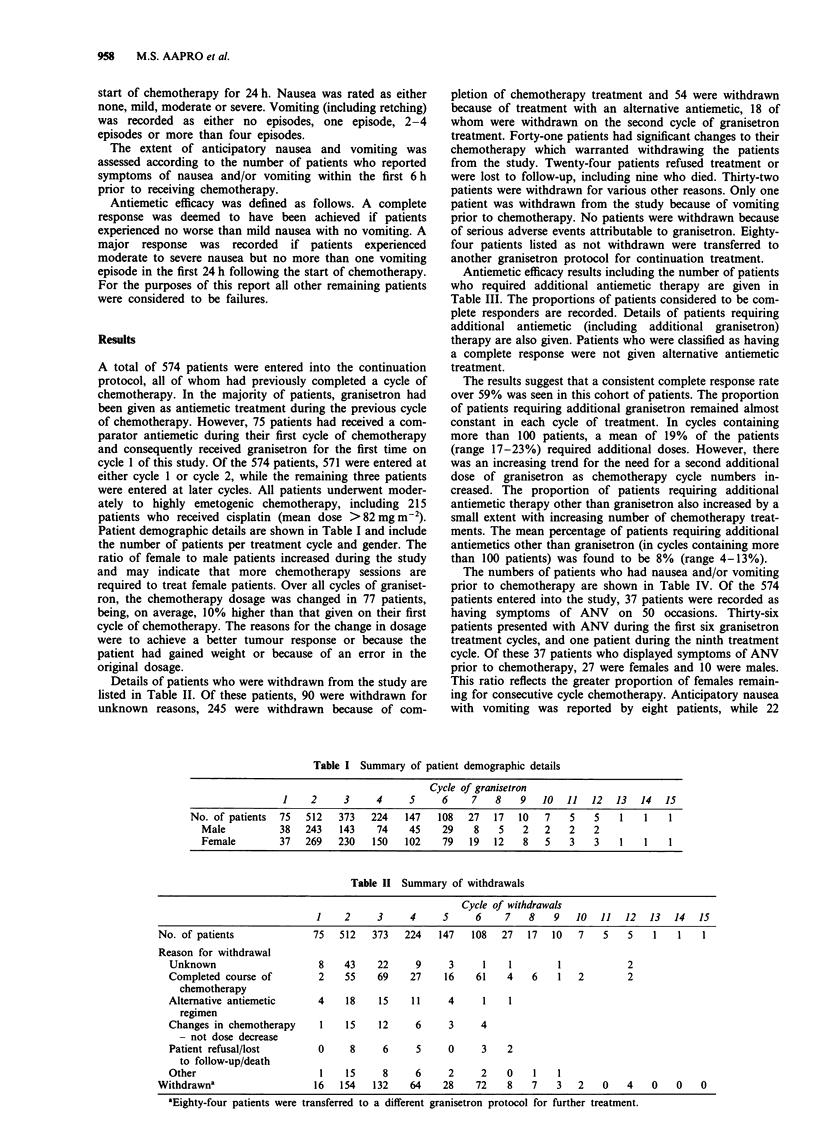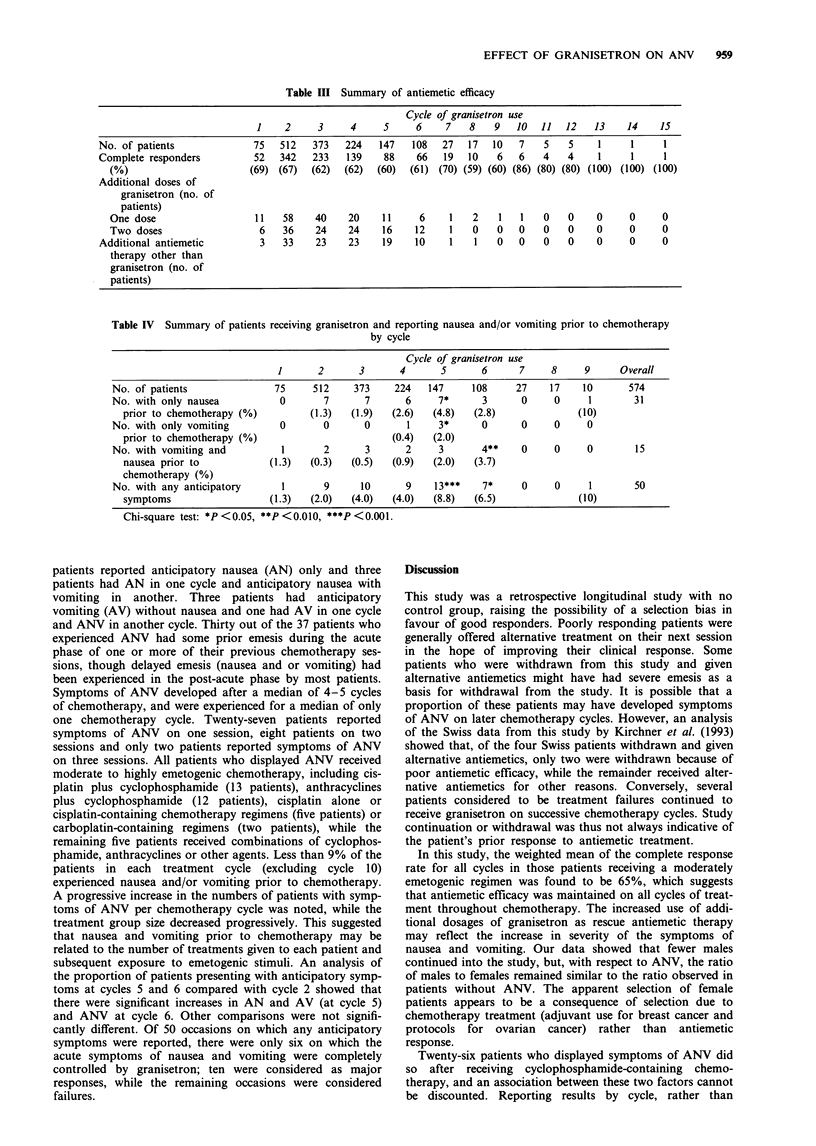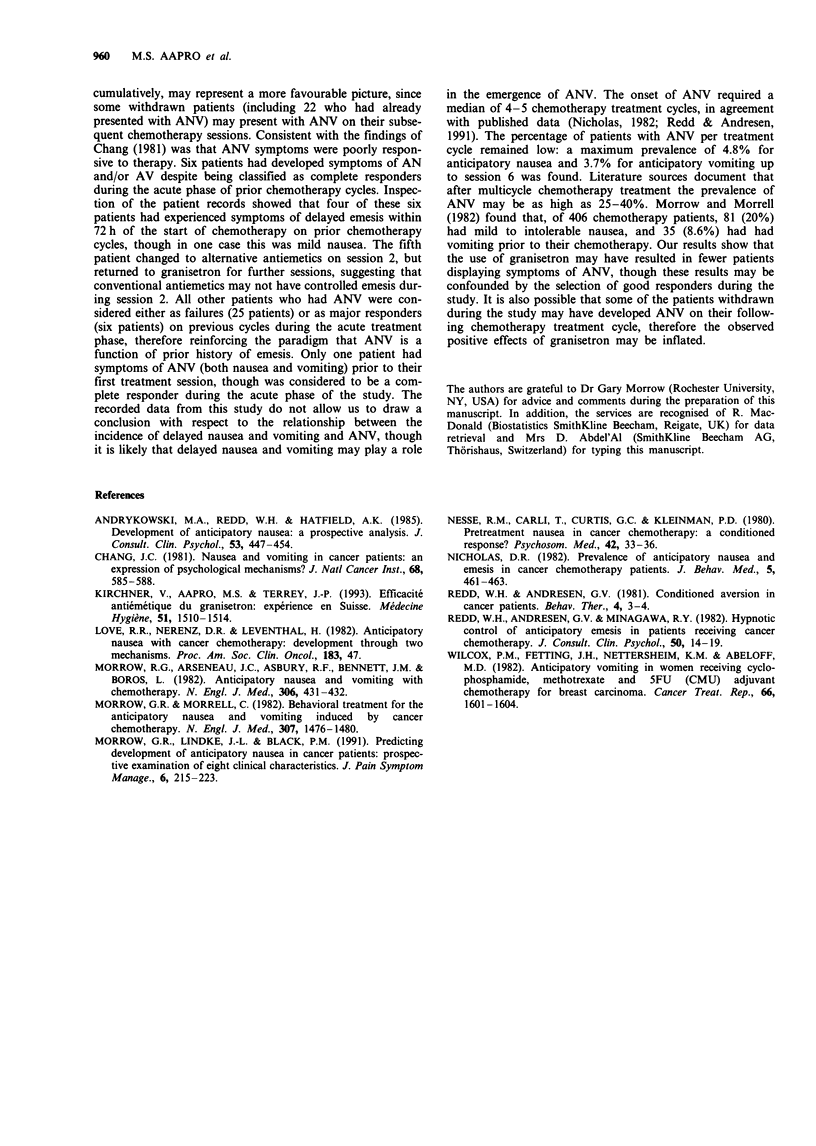# The incidence of anticipatory nausea and vomiting after repeat cycle chemotherapy: the effect of granisetron.

**DOI:** 10.1038/bjc.1994.185

**Published:** 1994-05

**Authors:** M. S. Aapro, V. Kirchner, J. P. Terrey

**Affiliations:** Centre Anticancéreux, Genolier, Switzerland.

## Abstract

Anticipatory nausea and vomiting (ANV) after repeated cycles of cytotoxic chemotherapy is thought to be a conditioned response to a conditioning stimulus. Good control of acute and delayed emesis may result in a lower incidence of ANV. We have analysed data from 574 chemotherapy patients who received granisetron as their antiemetic treatment during repeat cycle chemotherapy. Per treatment cycle, less than 10% of patients displayed symptoms of anticipatory nausea and 2% or less had symptoms of anticipatory vomiting. It is concluded that the use of granisetron as an antiemetic during the acute phase of chemotherapy may result in a lower incidence of ANV in patients undergoing repeat cycle chemotherapy.


Br. J. Cancer (1994), 69, 957–960 © Macmillan Press Ltd., 1994

### The incidence of anticipatory nausea and vomiting after repeat cycle chemotherapy: the effect of granisetron

M.S. Aapro[1,2], V. Kirchner[2] & J.P. Terrey[3]

[1]*Centre Anticancéreux, 1261 Genolier, Switzerland;* [2]*Hôpital Cantonal Universitaire Genève, 1211 Geneva, Switzerland;* [3]*SmithKline Beecham AG, 3174 Thörishaus, Switzerland.*

**Summary** Anticipatory nausea and vomiting (ANV) after repeated cycles of cytotoxic chemotherapy is thought to be a conditioned response to a conditioning stimulus. Good control of acute and delayed emesis may result in a lower incidence of ANV. We have analysed data from 574 chemotherapy patients who received granisetron as their antiemetic treatment during repeat cycle chemotherapy. Per treatment cycle, less than 10% of patients displayed symptoms of anticipatory nausea and 2% or less had symptoms of anticipatory vomiting. It is concluded that the use of granisetron as an antiemetic during the acute phase of chemotherapy may result in a lower incidence of ANV in patients undergoing repeat cycle chemotherapy.

The treatment of many forms of malignant disease often involves the use of cytotoxic agents whose toxic effects include nausea and/or vomiting. Andrykowski *et al.* (1985) showed that, in patients undergoing the initial phase of chemotherapy, emesis is an unconditioned response. However, after a few cycles of emetogenic chemotherapy during which the patient experiences emesis, the association of factors linked with chemotherapy (visual, gustatory, olfactory, environmental) may themselves initiate emesis. These factors are considered to be conditioned stimuli eliciting a conditioned response (emesis). Typically, these factors may precipitate emesis prior to the administration of chemotherapy, and this emesis is referred to as anticipatory nausea and vomiting (ANV). The incidence of ANV may vary widely from centre to centre owing to heterogeneity of patients and treatments. Early reviews of the prevalence of ANV showed that it ranges from 28.3% (Nicholas, 1982) to 44% (Nesse *et al.*, 1980). Love *et al.* (1982) reported a prevalence of anticipatory vomiting of 38%. The percentage of patients who reported ANV was subdivided by Morrow *et al.* (1982) into 24% who experienced anticipatory nausea and 9% anticipatory vomiting. The relationship between the development of ANV and the number of chemotherapy treatments has also been documented (Redd & Andresen, 1981; Nicholas, 1982; Redd *et al.*, 1982).

Chang (1981) showed that ANV is poorly responsive to treatment with conventional antiemetics, and much effort has been put into understanding the aetiology of ANV by Morrow *et al.* (1991). Behavioural treatment (Morrow & Morrell, 1982) to desensitise patients by means of counselling or by relaxation techniques has been considered.

It is now accepted that the development of ANV is a function of the emetogenicity and frequency of chemotherapy treatments. In a study by Wilcox *et al.* (1982), prevention of post-chemotherapy nausea and vomiting by conventional antiemetics was associated with the prevention of symptoms of ANV. The recent introduction of the potent and selective $5\text{-}HT_3$ antagonists as antiemetics (granisetron, ondansetron and tropisetron) has been regarded as being a significant advance in the control of acute emesis. In this paper we analyse the incidence of the symptoms of ANV of patients who have undergone repeat cycles of moderately emetogenic chemotherapy and who received granisetron as their antiemetic treatment.

### Materials and methods

Chemotherapy-naive patients who had given their informed consent were entered into one of several large multicentre, multinational antiemetic studies involving granisetron. Patients received granisetron under a continuation protocol for their successive chemotherapy treatment, provided that this did not change. This report relates to those patients treated within this continuation protocol. These antiemetic studies and the continuation protocol conformed to the Declaration of Helsinki (Hong Kong Amendment 1989) and were reviewed by the ethics review boards of each participating centre prior to the commencement of the study. A 10% variation in chemotherapy dose was allowed for changes in patient weight between cycles. The cytotoxic regimen had to include at least one of the following inravenous agents given as a single dose on day 1 of the cycle of chemotherapy: carboplatin $>300\ mg\ m^{-2}$; cisplatin $>20\ mg\ m^{-2}$; cyclophosphamide $>600\ mg\ m^{-2}$ i.v. when given in combination with other agents; dacarbazine $>350\ mg\ m^{-2}$ and $<500\ mg\ m^{-2}$; doxorubicin $>40\ mg\ m^{-2}$ as a single agent or $>25\ mg\ m^{-2}$ in combination with other cytotoxic agents; epirubicin $>75\ mg\ m^{-2}$ as single agent or $>50\ mg\ m^{-2}$ in combination with other agents; mustine $>6\ mg\ m^{-2}$. Other cytostatic agents were administered concurrently with one of the above chemotherapy agents.

Granisetron was administered 10 min before chemotherapy to each patient as a $40\ \mu g\ kg^{-1}$ intravenous infusion of 20 ml of 0.9% sodium chloride solution over 5 min. In the event of nausea and vomiting during the first 24 h, one or two (but not more) additional doses of granisetron ($40\ \mu g\ kg^{-1}$) could be given, at least 10 min apart. Standard antiemetics could be given if granisetron failed to control the symptoms of nausea and vomiting. Patients were entered into the trial if they satisfied the following criteria: they must have already completed a cycle of chemotherapy in a granisetron study and wish to receive granisetron treatment with subsequent cycles of the chemotherapy; a minimum age of 16 years; ability to comply with protocol procedures; and ability to give informed consent. Patients were excluded if they had suffered a serious adverse event suspected of being caused by granisetron; if they had marked hepatic or renal dysfunction, an active peptic ulcer, gastric compression or tumour involvement likely to lead to subacute obstruction; if they had received any other investigational drug within the past 3 months or were due to receive such drugs during this cycle of chemotherapy; if there had been changes in their chemotherapy other than a dose reduction.

Patients were allowed to continue to take previous CNS medication but not to start treatment with these drugs because of their amnesic effects. They were all hospitalised for a minimum period of 2 h following the administration of their chemotherapy. Clinical and laboratory monitoring was carried out in the patients immediately before, during and after their chemotherapy treatment.

Subjective assessments of acute nausea and vomiting were made retrospectively every 6 h by the patient beginning at the

Correspondence: M.S. Aapro.
Received 16 June 1993; and in revised form 6 January 1994.

start of chemotherapy for 24 h. Nausea was rated as either none, mild, moderate or severe. Vomiting (including retching) was recorded as either no episodes, one episode, 2–4 episodes or more than four episodes.

The extent of anticipatory nausea and vomiting was assessed according to the number of patients who reported symptoms of nausea and/or vomiting within the first 6 h prior to receiving chemotherapy.

Antiemetic efficacy was defined as follows. A complete response was deemed to have been achieved if patients experienced no worse than mild nausea with no vomiting. A major response was recorded if patients experienced moderate to severe nausea but no more than one vomiting episode in the first 24 h following the start of chemotherapy. For the purposes of this report all other remaining patients were considered to be failures.

### Results

A total of 574 patients were entered into the continuation protocol, all of whom had previously completed a cycle of chemotherapy. In the majority of patients, granisetron had been given as antiemetic treatment during the previous cycle of chemotherapy. However, 75 patients had received a comparator antiemetic during their first cycle of chemotherapy and consequently received granisetron for the first time on cycle 1 of this study. Of the 574 patients, 571 were entered at either cycle 1 or cycle 2, while the remaining three patients were entered at later cycles. All patients underwent moderately to highly emetogenic chemotherapy, including 215 patients who received cisplatin (mean dose $>82\ mg\ m^{-2}$). Patient demographic details are shown in Table I and include the number of patients per treatment cycle and gender. The ratio of female to male patients increased during the study and may indicate that more chemotherapy sessions are required to treat female patients. Over all cycles of granisetron, the chemotherapy dosage was changed in 77 patients, being, on average, 10% higher than that given on their first cycle of chemotherapy. The reasons for the change in dosage were to achieve a better tumour response or because the patient had gained weight or because of an error in the original dosage.

Details of patients who were withdrawn from the study are listed in Table II. Of these patients, 90 were withdrawn for unknown reasons, 245 were withdrawn because of completion of chemotherapy treatment and 54 were withdrawn because of treatment with an alternative antiemetic, 18 of whom were withdrawn on the second cycle of granisetron treatment. Forty-one patients had significant changes to their chemotherapy which warranted withdrawing the patients from the study. Twenty-four patients refused treatment or were lost to follow-up, including nine who died. Thirty-two patients were withdrawn for various other reasons. Only one patient was withdrawn from the study because of vomiting prior to chemotherapy. No patients were withdrawn because of serious adverse events attributable to granisetron. Eighty-four patients listed as not withdrawn were transferred to another granisetron protocol for continuation treatment.

Antiemetic efficacy results including the number of patients who required additional antiemetic therapy are given in Table III. The proportions of patients considered to be complete responders are recorded. Details of patients requiring additional antiemetic (including additional granisetron) therapy are also given. Patients who were classified as having a complete response were not given alternative antiemetic treatment.

The results suggest that a consistent complete response rate over 59% was seen in this cohort of patients. The proportion of patients requiring additional granisetron remained almost constant in each cycle of treatment. In cycles containing more than 100 patients, a mean of 19% of the patients (range 17–23%) required additional doses. However, there was an increasing trend for the need for a second additional dose of granisetron as chemotherapy cycle numbers increased. The proportion of patients requiring additional antiemetic therapy other than granisetron also increased by a small extent with increasing number of chemotherapy treatments. The mean percentage of patients requiring additional antiemetics other than granisetron (in cycles containing more than 100 patients) was found to be 8% (range 4–13%).

The numbers of patients who had nausea and/or vomiting prior to chemotherapy are shown in Table IV. Of the 574 patients entered into the study, 37 patients were recorded as having symptoms of ANV on 50 occasions. Thirty-six patients presented with ANV during the first six granisetron treatment cycles, and one patient during the ninth treatment cycle. Of these 37 patients who displayed symptoms of ANV prior to chemotherapy, 27 were females and 10 were males. This ratio reflects the greater proportion of females remaining for consecutive cycle chemotherapy. Anticipatory nausea with vomiting was reported by eight patients, while 22

**Table I** Summary of patient demographic details

| | *Cycle of granisetron* | | | | | | | | | | | | | | |
|---|---|---|---|---|---|---|---|---|---|---|---|---|---|---|---|
| | *1* | *2* | *3* | *4* | *5* | *6* | *7* | *8* | *9* | *10* | *11* | *12* | *13* | *14* | *15* |
| No. of patients | 75 | 512 | 373 | 224 | 147 | 108 | 27 | 17 | 10 | 7 | 5 | 5 | 1 | 1 | 1 |
| Male | 38 | 243 | 143 | 74 | 45 | 29 | 8 | 5 | 2 | 2 | 2 | 2 | | | |
| Female | 37 | 269 | 230 | 150 | 102 | 79 | 19 | 12 | 8 | 5 | 3 | 3 | 1 | 1 | 1 |

**Table II** Summary of withdrawals

| | *Cycle of withdrawals* | | | | | | | | | | | | | | |
|---|---|---|---|---|---|---|---|---|---|---|---|---|---|---|---|
| | *1* | *2* | *3* | *4* | *5* | *6* | *7* | *8* | *9* | *10* | *11* | *12* | *13* | *14* | *15* |
| No. of patients | 75 | 512 | 373 | 224 | 147 | 108 | 27 | 17 | 10 | 7 | 5 | 5 | 1 | 1 | 1 |
| Reason for withdrawal | | | | | | | | | | | | | | | |
| Unknown | 8 | 43 | 22 | 9 | 3 | 1 | 1 | | 1 | | | 2 | | | |
| Completed course of chemotherapy | 2 | 55 | 69 | 27 | 16 | 61 | 4 | 6 | 1 | 2 | | 2 | | | |
| Alternative antiemetic regimen | 4 | 18 | 15 | 11 | 4 | 1 | 1 | | | | | | | | |
| Changes in chemotherapy – not dose decrease | 1 | 15 | 12 | 6 | 3 | 4 | | | | | | | | | |
| Patient refusal/lost to follow-up/death | 0 | 8 | 6 | 5 | 0 | 3 | 2 | | | | | | | | |
| Other | 1 | 15 | 8 | 6 | 2 | 2 | 0 | 1 | 1 | | | | | | |
| Withdrawn[a] | 16 | 154 | 132 | 64 | 28 | 72 | 8 | 7 | 3 | 2 | 0 | 4 | 0 | 0 | 0 |

[a]Eighty-four patients were transferred to a different granisetron protocol for further treatment.

**Table III** Summary of antiemetic efficacy

| | Cycle of granisetron use | | | | | | | | | | | | | | |
|---|---|---|---|---|---|---|---|---|---|---|---|---|---|---|---|
| | *1* | *2* | *3* | *4* | *5* | *6* | *7* | *8* | *9* | *10* | *11* | *12* | *13* | *14* | *15* |
| No. of patients | 75 | 512 | 373 | 224 | 147 | 108 | 27 | 17 | 10 | 7 | 5 | 5 | 1 | 1 | 1 |
| Complete responders (%) | 52 (69) | 342 (67) | 233 (62) | 139 (62) | 88 (60) | 66 (61) | 19 (70) | 10 (59) | 6 (60) | 6 (86) | 4 (80) | 4 (80) | 1 (100) | 1 (100) | 1 (100) |
| Additional doses of granisetron (no. of patients) | | | | | | | | | | | | | | | |
| One dose | 11 | 58 | 40 | 20 | 11 | 6 | 1 | 2 | 1 | 1 | 0 | 0 | 0 | 0 | 0 |
| Two doses | 6 | 36 | 24 | 24 | 16 | 12 | 1 | 0 | 0 | 0 | 0 | 0 | 0 | 0 | 0 |
| Additional antiemetic therapy other than granisetron (no. of patients) | 3 | 33 | 23 | 23 | 19 | 10 | 1 | 1 | 0 | 0 | 0 | 0 | 0 | 0 | 0 |

**Table IV** Summary of patients receiving granisetron and reporting nausea and/or vomiting prior to chemotherapy by cycle

| | Cycle of granisetron use | | | | | | | | | |
|---|---|---|---|---|---|---|---|---|---|---|
| | *1* | *2* | *3* | *4* | *5* | *6* | *7* | *8* | *9* | *Overall* |
| No. of patients | 75 | 512 | 373 | 224 | 147 | 108 | 27 | 17 | 10 | 574 |
| No. with only nausea prior to chemotherapy (%) | 0 | 7 (1.3) | 7 (1.9) | 6 (2.6) | 7* (4.8) | 3 (2.8) | 0 | 0 | 1 (10) | 31 |
| No. with only vomiting prior to chemotherapy (%) | 0 | 0 | 0 | 1 (0.4) | 3* (2.0) | 0 | 0 | 0 | 0 | |
| No. with vomiting and nausea prior to chemotherapy (%) | 1 (1.3) | 2 (0.3) | 3 (0.5) | 2 (0.9) | 3 (2.0) | 4** (3.7) | 0 | 0 | 0 | 15 |
| No. with any anticipatory symptoms | 1 (1.3) | 9 (2.0) | 10 (4.0) | 9 (4.0) | 13*** (8.8) | 7* (6.5) | 0 | 0 | 1 (10) | 50 |

Chi-square test: $^{*}P < 0.05$, $^{**}P < 0.010$, $^{***}P < 0.001$.

patients reported anticipatory nausea (AN) only and three patients had AN in one cycle and anticipatory nausea with vomiting in another. Three patients had anticipatory vomiting (AV) without nausea and one had AV in one cycle and ANV in another cycle. Thirty out of the 37 patients who experienced ANV had some prior emesis during the acute phase of one or more of their previous chemotherapy sessions, though delayed emesis (nausea and or vomiting) had been experienced in the post-acute phase by most patients. Symptoms of ANV developed after a median of 4–5 cycles of chemotherapy, and were experienced for a median of only one chemotherapy cycle. Twenty-seven patients reported symptoms of ANV on one session, eight patients on two sessions and only two patients reported symptoms of ANV on three sessions. All patients who displayed ANV received moderate to highly emetogenic chemotherapy, including cisplatin plus cyclophosphamide (13 patients), anthracyclines plus cyclophosphamide (12 patients), cisplatin alone or cisplatin-containing chemotherapy regimens (five patients) or carboplatin-containing regimens (two patients), while the remaining five patients received combinations of cyclophosphamide, anthracyclines or other agents. Less than 9% of the patients in each treatment cycle (excluding cycle 10) experienced nausea and/or vomiting prior to chemotherapy. A progressive increase in the numbers of patients with symptoms of ANV per chemotherapy cycle was noted, while the treatment group size decreased progressively. This suggested that nausea and vomiting prior to chemotherapy may be related to the number of treatments given to each patient and subsequent exposure to emetogenic stimuli. An analysis of the proportion of patients presenting with anticipatory symptoms at cycles 5 and 6 compared with cycle 2 showed that there were significant increases in AN and AV (at cycle 5) and ANV at cycle 6. Other comparisons were not significantly different. Of 50 occasions on which any anticipatory symptoms were reported, there were only six on which the acute symptoms of nausea and vomiting were completely controlled by granisetron; ten were considered as major responses, while the remaining occasions were considered failures.

### Discussion

This study was a retrospective longitudinal study with no control group, raising the possibility of a selection bias in favour of good responders. Poorly responding patients were generally offered alternative treatment on their next session in the hope of improving their clinical response. Some patients who were withdrawn from this study and given alternative antiemetics might have had severe emesis as a basis for withdrawal from the study. It is possible that a proportion of these patients may have developed symptoms of ANV on later chemotherapy cycles. However, an analysis of the Swiss data from this study by Kirchner *et al.* (1993) showed that, of the four Swiss patients withdrawn and given alternative antiemetics, only two were withdrawn because of poor antiemetic efficacy, while the remainder received alternative antiemetics for other reasons. Conversely, several patients considered to be treatment failures continued to receive granisetron on successive chemotherapy cycles. Study continuation or withdrawal was thus not always indicative of the patient's prior response to antiemetic treatment.

In this study, the weighted mean of the complete response rate for all cycles in those patients receiving a moderately emetogenic regimen was found to be 65%, which suggests that antiemetic efficacy was maintained on all cycles of treatment throughout chemotherapy. The increased use of additional dosages of granisetron as rescue antiemetic therapy may reflect the increase in severity of the symptoms of nausea and vomiting. Our data showed that fewer males continued into the study, but, with respect to ANV, the ratio of males to females remained similar to the ratio observed in patients without ANV. The apparent selection of female patients appears to be a consequence of selection due to chemotherapy treatment (adjuvant use for breast cancer and protocols for ovarian cancer) rather than antiemetic response.

Twenty-six patients who displayed symptoms of ANV did so after receiving cyclophosphamide-containing chemotherapy, and an association between these two factors cannot be discounted. Reporting results by cycle, rather than cumulatively, may represent a more favourable picture, since some withdrawn patients (including 22 who had already presented with ANV) may present with ANV on their subsequent chemotherapy sessions. Consistent with the findings of Chang (1981) was that ANV symptoms were poorly responsive to therapy. Six patients had developed symptoms of AN and/or AV despite being classified as complete responders during the acute phase of prior chemotherapy cycles. Inspection of the patient records showed that four of these six patients had experienced symptoms of delayed emesis within 72 h of the start of chemotherapy on prior chemotherapy cycles, though in one case this was mild nausea. The fifth patient changed to alternative antiemetics on session 2, but returned to granisetron for further sessions, suggesting that conventional antiemetics may not have controlled emesis during session 2. All other patients who had ANV were considered either as failures (25 patients) or as major responders (six patients) on previous cycles during the acute treatment phase, therefore reinforcing the paradigm that ANV is a function of prior history of emesis. Only one patient had symptoms of ANV (both nausea and vomiting) prior to their first treatment session, though was considered to be a complete responder during the acute phase of the study. The recorded data from this study do not allow us to draw a conclusion with respect to the relationship between the incidence of delayed nausea and vomiting and ANV, though it is likely that delayed nausea and vomiting may play a role in the emergence of ANV. The onset of ANV required a median of 4–5 chemotherapy treatment cycles, in agreement with published data (Nicholas, 1982; Redd & Andresen, 1991). The percentage of patients with ANV per treatment cycle remained low: a maximum prevalence of 4.8% for anticipatory nausea and 3.7% for anticipatory vomiting up to session 6 was found. Literature sources document that after multicycle chemotherapy treatment the prevalence of ANV may be as high as 25–40%. Morrow and Morrell (1982) found that, of 406 chemotherapy patients, 81 (20%) had mild to intolerable nausea, and 35 (8.6%) had had vomiting prior to their chemotherapy. Our results show that the use of granisetron may have resulted in fewer patients displaying symptoms of ANV, though these results may be confounded by the selection of good responders during the study. It is also possible that some of the patients withdrawn during the study may have developed ANV on their following chemotherapy treatment cycle, therefore the observed positive effects of granisetron may be inflated.

The authors are grateful to Dr Gary Morrow (Rochester University, NY, USA) for advice and comments during the preparation of this manuscript. In addition, the services are recognised of R. MacDonald (Biostatistics SmithKline Beecham, Reigate, UK) for data retrieval and Mrs D. Abdel'Al (SmithKline Beecham AG, Thörishaus, Switzerland) for typing this manuscript.

### References

ANDRYKOWSKI, M.A., REDD, W.H. & HATFIELD, A.K. (1985). Development of anticipatory nausea: a prospective analysis. *J. Consult. Clin. Psychol.*, **53**, 447–454.

CHANG, J.C. (1981). Nausea and vomiting in cancer patients: an expression of psychological mechanisms? *J. Natl Cancer Inst.*, **68**, 585–588.

KIRCHNER, V., AAPRO, M.S. & TERREY, J.-P. (1993). Efficacité antiémétique du granisetron: expérience en Suisse. *Médecine Hygiène*, **51**, 1510–1514.

LOVE, R.R., NERENZ, D.R. & LEVENTHAL, H. (1982). Anticipatory nausea with cancer chemotherapy: development through two mechanisms. *Proc. Am. Soc. Clin. Oncol.*, **183**, 47.

MORROW, R.G., ARSENEAU, J.C., ASBURY, R.F., BENNETT, J.M. & BOROS, L. (1982). Anticipatory nausea and vomiting with chemotherapy. *N. Engl. J. Med.*, **306**, 431–432.

MORROW, G.R. & MORRELL, C. (1982). Behavioral treatment for the anticipatory nausea and vomiting induced by cancer chemotherapy. *N. Engl. J. Med.*, **307**, 1476–1480.

MORROW, G.R., LINDKE, J.-L. & BLACK, P.M. (1991). Predicting development of anticipatory nausea in cancer patients: prospective examination of eight clinical characteristics. *J. Pain Symptom Manage.*, **6**, 215–223.

NESSE, R.M., CARLI, T., CURTIS, G.C. & KLEINMAN, P.D. (1980). Pretreatment nausea in cancer chemotherapy: a conditioned response? *Psychosom. Med.*, **42**, 33–36.

NICHOLAS, D.R. (1982). Prevalence of anticipatory nausea and emesis in cancer chemotherapy patients. *J. Behav. Med.*, **5**, 461–463.

REDD, W.H. & ANDRESEN, G.V. (1981). Conditioned aversion in cancer patients. *Behav. Ther.*, **4**, 3–4.

REDD, W.H., ANDRESEN, G.V. & MINAGAWA, R.Y. (1982). Hypnotic control of anticipatory emesis in patients receiving cancer chemotherapy. *J. Consult. Clin. Psychol.*, **50**, 14–19.

WILCOX, P.M., FETTING, J.H., NETTERSHEIM, K.M. & ABELOFF, M.D. (1982). Anticipatory vomiting in women receiving cyclophosphamide, methotrexate and 5FU (CMU) adjuvant chemotherapy for breast carcinoma. *Cancer Treat. Rep.*, **66**, 1601–1604.